# Gaining role clarity in working with sick leave questions—Registered Nurses’ experiences of an educational intervention

**DOI:** 10.1002/nop2.201

**Published:** 2018-09-14

**Authors:** Linda Lännerström, Thorne Wallman, Elenor Kaminsky, Inger K Holmström

**Affiliations:** ^1^ Department of Public Health and Caring Sciences, Family Medicine and Preventive Medicine Section Uppsala University Uppsala Sweden; ^2^ Centre for Clinical Research Sörmland Uppsala University Eskilstuna Sweden; ^3^ School of Health, Care and Social Welfare Mälardalen University Västerås Sweden; ^4^ Department of Public Health and Caring Sciences, Health Services Research Section Uppsala University Uppsala Sweden

**Keywords:** cluster randomized controlled study, educational intervention, interviews, sick leave, social insurance medicine, telephone nursing

## Abstract

**Aim:**

To describe how a short educational intervention in social insurance medicine was experienced by Registered Nurses and what changes it brought to their work with sick leave questions in telephone nursing.

**Design:**

Qualitative explorative interview study.

**Methods:**

Interviews with 12 purposively sampled Registered Nurses were conducted and analysed using manifest content analysis.

**Results:**

The intervention increased Registered Nurses’ knowledge of the sick leave process and changed their work habits as they now have more of the skills needed to handle sick leave questions. In this way, they gained role clarity in their work with sick leave questions. The new knowledge included rules and regulations, actors’ roles and patients’ experiences. Learning from peers, reflecting and having the opportunity to ask questions were also described as increasing their knowledge. The skills following the participation were described as knowing what to say and do and knowing where to turn for support.

## 1 BACKGROUND

Registered Nurses (RNs) are not commonly considered to have a role related to the work with sick leave in primary healthcare settings. However, recent research using data from one Swedish county indicates that RNs do have work tasks connected to sick leave as they handle telephone calls concerning sick leave questions (SLQs), that is questions related to the social insurance regulations and sickness certification (Lännerström, von Celsing, Holmström, & Wallman, 2017). Consequently, telephone calls are a channel for people’s SLQs, and the RN’s role is to handle those calls, hence, they take part in the sick leave process by handling calls in telephone nursing. Here, the “sick leave process” is defined as the process initiated when a person cannot work due to sickness and reports sick. In a study from 2012 by Lännerström, Wallman and Söderback, RNs described their work with SLQs as including, first, making an assessment of the appropriate action, followed by one or several of the following: making an appointment, giving information and guidance to the patient and/or monitoring the patient’s rights.

As in nursing in general, the goal of telephone nursing is to promote health, prevent illness, restore health and alleviate suffering by identifying and meeting the care‐seeker’s needs (Greenberg, 2009; Rutenberg & Greenberg, 2012). In telephone nursing, the nursing process includes collecting information; assessing care needs; making decisions; giving advice, support and education; referring patients to the optimal level of care; giving information; and coordinating care (Greenberg, 2009; Kaminsky, 2013; Rutenberg & Greenberg, 2012). Telephone nursing is a challenging task that demands high competence for making appropriate assessments and decisions (Greenberg, 2009; Kaminsky, 2013; Rutenberg & Greenberg, 2012; Wahlberg, Cedersund, & Wredling, 2003). Telephone nursing in a primary healthcare setting is especially demanding, as it requires not only a broad general knowledge of nursing, communication, pedagogy and medicine, but also of local routines and organizational responsibilities (Kaminsky, Rosenqvist, & Holmström, 2009; Swedish Society of Nursing, 2011b). Handling SLQs demands additional knowledge of social insurance medicine. Handling callers’ SLQs has, in our previous studies, been highlighted as a problematic task in telephone nursing as the experience is linked to a lack of education in social insurance medicine and the skills for handling these questions—the “know‐how” (Lännerström, Celsing, et al., 2017; Lännerström, Holmström, Svärdsudd, & Wallman, 2017; Lännerström, Wallman, & Söderback, 2012). Therefore, the education and training of RNs working with SLQs is essential. In a previous Swedish study, by Müssener and Linderoth (2009), RNs working with SLQs via telephone described having increased their knowledge regarding social insurance medicine after a two‐day training session on this matter. The study further highlighted that the participating RNs found it easier after the intervention to ask questions that stimulated patients’ own sense of responsibility and to refer them to other professionals besides physicians.

To our knowledge, only one other study of RNs’ work with SLQs in telephone nursing (Müssener & Linderoth, 2009) has been published apart from the work made by the present research group. Thus, research in this area is very sparse. The knowledge gap is large and embraces both descriptions of RNs’ practical work regarding SLQs in telephone nursing and also how this practice can be developed. Knowledge is also lacking regarding effects interventions have on practice, in relation to both RNs and patients.

To enhance RNs’ professional competence to handle SLQs, an educational intervention was conducted. The effect of the intervention will be presented in an upcoming paper (in manuscript) describing that the intervention seemed to have a strong effect on RNs experiencing fewer problems with handling SLQs. However, the overall effect was inconclusive and needs further examination in larger samples. The present paper reports on the process evaluation, as experienced by the RNs participating in the intervention.

With the above in mind, the aim of this study was to describe how a short educational intervention in social insurance medicine was experienced by RNs and what changes it brought to their work with sick leave questions in telephone nursing.

The research questions were as follows: “How can participation in an educational intervention in social insurance medicine be experienced by RNs?” and “How is an educational intervention in social insurance medicine experienced to change the way of working in telephone nursing?”

## 2 THEORETICAL FRAMEWORK

Continuous competence development is important for the nursing profession (Bindon, 2017; EFN, 2015). The intervention reported in the present study, which aimed to enhance RNs’ competence in handling SLQs, was based on theories of competence development and reflection. How competence is achieved and developed is dependent on different epistemological premises in different sciences. In human science, where nursing has its roots, competence development is grounded in the notion that humans are active, creative and inseparable from context and the world (ICN, 2012; Swedish Society of Nursing, 2011a). Competence development thus needs to address and make use of humans’ inherent creativity and activity. In nursing, reflection is often used as a method for enhancing competence by making use of this same inherent human activity and creativity (further described later; Aglen, 2016; Benner, 1984; Dall’Alba & Sandberg, 2006; Kuiper & Pesut, 2004; Ruth‐Sahd, 2003; Schön, 1987, 1995 ). Outcomes of clinical practice reflection include aspects such as increased learning from experience, the acceptance of professional responsibility and continual professional growth and improvement in practice by promoting self‐awareness (Ruth‐Sahd, 2003). Here, *education* is defined as both the education and the training offered at universities and the “in‐house” local educational interventions offered at RNs’ workplaces to develop their competence. Furthermore, *competence* is defined here as a combination of attributes (i.e., knowledge, skills, attitudes and understanding of work) applied in tasks carried out in professional practice (Dall’Alba & Sandberg, 1996; EFN, 2015; Schön, 1987, 1995 ).

Internationally, in nursing, competence development through reflection is often connected to Schön’s theory of *the reflective practitioner* (1987, 1995). He argues that it is not merely through higher, scientifically based education that competence in handling practical tasks can be achieved; it also needs to be developed through real‐world practice and the clinical context. In those settings, problems are often complex, uncertain, and unstable and involve value conflicts that do not fit existing theories. In the reflective in‐/on‐/for‐action paradigm inherent in learning by doing and coaching, professionals can achieve higher and more developed levels of competence.

Nursing researcher Patricia Benner describes RNs’ competence development in a similar way, as that of moving from novice to expert. According to her, based on Heidegger (1962) and Gadamer (1979), experience emerges when preconceived opinions and expectations are challenged, improved and/or falsified in practice. The “intuitive” processes in clinical decision‐making are difficult to describe; however, they are tacit (derived from Polanyi & Sen, 2009). Nonetheless, they can be described through interpretive descriptions in clinical practice (Benner, 1984; Miraglia & Asselin, 2015). Thus, by describing practice, learning can be enhanced. Sandberg’s work adds another dimension to competence development, emphasizing that professionals’ understanding of their work is crucial for how the work is performed (Dall’Alba & Sandberg, 1996; Sandberg, 2001). This understanding guides how the worker takes on and develops his or her work assignments. Sandberg argues that designing educational interventions to address workers’ understanding of their work helps them to achieve a higher level of competence. This notion is based on the concept of phenomenological philosophy and specifically refers to Husserl (Husserl & Carr, 1978), Schuetz (1945, 1953 ) and Berger and Luckmann (1991). In phenomenology, understanding is created in everyday life when the mind is exposed to phenomena and gives meaning to these phenomena. When applying this to work, the understanding of one’s work is constituted of the given meaning it has for the worker. Sandberg, together with Dall'Alba, argues that, by using the tool of reflection, the structure of meaning can change, and understanding is altered. This includes not only acquiring new knowledge, but also experiencing aspects of, in this case, the care practice in a different way. The most effective way to develop meaning is by alternating between experiencing aspects of practice and the practice as a whole. In this way, the worker develops an understanding of not only what to do but also of what it is to be a professional (Dall’Alba & Sandberg, 1996). From this point of view, which is also the approach taken in the present intervention study, professional competence is developed gradually in the meaning‐making of theoretical knowledge and clinical problem‐solving when reflecting.

## 3 METHODS

### 3.1 Design and sample

The present study is a qualitative explorative interview study to evaluate a cluster randomized controlled educational intervention. Twelve RNs who had participated in the intervention agreed to take part in telephone interviews (Table 1). The intervention was performed in 2014 in a county in central Sweden. RNs from eleven centres were invited to participate and 28 of the 59 eligible RNs completed the intervention. Of those 28 RNs, 12 were purposively sampled (Polit & Beck, 2012) for interviews in the present study.

**Table 1:** Characteristics of the participants

Age	Gender	Primary healthcare centre	Specialist nurse education	Experience of telephone nursing, years	Ownership primary healthcare centre	Employment	Working hours in telephone nursing, %	Education in social insurance medicine
30	Male	7	No	1–5	Public	Full‐time	50–100	No
58	Female	7	Yes	1–5	Public	Full‐time	50–100	No
51	Female	7	Yes	≥10	Public	Full‐time	50–100	No
46	Female	13	Yes	1–5	Public	Full‐time	<50	No
32	Male	2	No	1–5	Private	Full‐time	50–100	Missing
30	Male	2	Yes	1–5	Private	Full‐time	50–100	No
51	Female	2	Yes	≥10	Private	Part‐time	<50	No
35	Female	13	No	1–5	Public	Full‐time	50–100	No
65	Female	8	Yes	≥10	Public	Full‐time	50–100	No
60	Female	8	Yes	≥10	Public	Full‐time	50–100	No
45	Female	8	Yes	6–9	Public	Full‐time	50–100	No
55	Female	11	No	≥10	Public	Part‐time	50–100	No

### 3.2 Procedure

At the time of the intervention, the RNs’ managers agreed to allow them to participate in interviews during working hours. The RNs were invited by email to participate and were included after providing their informed consent. The interviews were performed by author EK, who had not been involved in the intervention but is a nursing researcher with long experience of interviewing, telephone nursing research and clinical telephone nursing. The interviews began with EK informing the participants of her qualifications. The interview guide was semi‐structured (Table 2) and questions included examples of the participants’ use of knowledge learnt during the intervention, whether and how it had influenced their daily work with SLQs and their suggestions for educational improvements. The interviews lasted between 14‐25 min were conducted by telephone and were recorded digitally.

**Table 2:** Interview guide

1	Tell me about your experience of attending the educational intervention in social insurance medicine
2	What was good about the education?
3	Give some examples of when you’ve had direct use of the education in your work
4	What are your concrete suggestions for improving the educational intervention in social insurance medicine?
5	In what way has the educational intervention in social insurance medicine changed the way you work with sick leave questions in telephone nursing?
6	Is there something else you want to say about the content and presentation of the intervention, or anything else you want to say?

### 3.3 Ethical considerations

The ethical principles for medical research involving human subjects outlined in the Declaration of Helsinki (World Medical Association, 2013) were followed. The participants received written information and a consent form by email. They were informed verbally that their participation was voluntary and could be withdrawn at any time. The findings are presented at group level, to assure confidentiality. Research Ethics Committee approval was granted by the Regional Ethics Review Board (Dnr 2014/156/1).

### 3.4 Intervention

The Committee of Social Insurance Medicine in the participating county council commissioned one of the researchers to educate RNs about social insurance medicine. The educational intervention was performed by two researchers (a telephone nurse and a rehabilitation medicine specialist physician), an official from the Swedish Social Insurance Agency and a healthcare official working with the regulation of social insurance medicine. When designing the intervention, a combination of lectures and reflection was used to connect theoretical knowledge with examples from the participants’ clinical practice. The decision to design the intervention in this way was guided by the previously described theoretical framework. According to a review by Grimshaw et al. (2001), there is no overall solution for ensuring that an intervention will have a great impact but, generally, active methods are more likely to affect behavioural change than passive approaches. Other studies have reported positive outcomes on professional practice and healthcare outcomes when interprofessional education is used (Dall’Alba & Sandberg, 1996).

The intervention included lectures about what RNs could do when handling SLQs, the physician’s role, laws and regulations, the assignment of the Social Insurance Agency and risk factors associated with sick leave. The RNs participated during ordinary working hours, which limited the intervention time; for this reason, education sessions were held on two half‐days (4 hr each), with one month in between. During Session 1, participants were asked to complete an assignment during the month before the next half‐day of education. The assignment included describing and reflecting, in writing, on a telephone call where the RN had handled a sick leave question. These reflections were then used as a part of group discussions during Session 2. Slightly more than half the course time consisted of teacher‐led group discussions.

### 3.5 Analysis

All interviews were transcribed verbatim. The texts were analysed using manifest content analysis, as described by Graneheim and Lundman (2004), to find differences and similarities in the described experiences. An inductive approach with no predetermined categories was used. Since the study aim was twofold, the analysis had two domains separating the experience of the intervention and the effect of the intervention in terms of changes in handling SLQs. All interviews were read several times, to give researchers an overall view and to familiarize with the content. After this, meaning units corresponding to the aim were sought and marked in the text. The units were condensed and coded, and the codes were sorted into categories. Among the categories, there were sub‐categories. After this, the categories and sub‐categories were checked against meaning units for concordance. A theme describing the underlying experience running through the categories emerged (Table 3). Authors LL and IKH performed the initial analysis and all authors then discussed and consented to the analysis.

**Table 3:** Domains, categories and sub‐categories regarding RNs experiences of the educational intervention in social insurance medicine and what changes it brought to their work with sick leave questions in telephone nursing

Domain	Sub‐category	Category	Theme
Experience of the intervention	Gaining knowledge of rules and regulations	Gaining knowledge of the sick leave process	
	Gaining knowledge of actors' different roles		
	Gaining knowledge of the patient’s perspective of sick leave		
	Learning from peers in group discussions		Gaining role clarity in working with SLQs
	Learning by reflecting and asking questions		
Experience of changes in handling	Knowing what to say and do	Having skills to handle SLQs	
SLQs	Knowing where to turn for support		

Note

SLQ: sick leave question.

## 4 FINDINGS

The main finding, the theme that emerged, was that the RNs *gained role clarity in their work with SLQs*. The findings are presented in the domains *Experience of intervention* and *Experience of* c*hanges in handling SLQs.* Each domain has underlying categories and sub‐categories, described in the following text (Table 3 for an overview of the analysis structure).

### 4.1 Domain: Experience of the intervention

The domain *Experience of the intervention* was described as the category *Gaining knowledge of the sick leave process,* with sub‐categories *Gaining knowledge of rules and regulations*, *Gaining knowledge of the patient’s perspective of sick leave*, *Learning from peers in group discussions* and *Learning by reflection and asking questions*.

### 4.2 Category: Gaining increased knowledge of the sick leave process

This category includes the participating RNs’ descriptions of increasing their knowledge of social insurance medicine and the sick leave process. Almost all descriptions of experiences of participating in the intervention referred to gaining increased knowledge. All RNs described their participation as a positive experience, although negative feedback was expressed in criticism regarding the shortness of time. Many of them described previously lacking the now‐attained knowledge. One RN described initially being highly sceptical of the intervention and fearing the participation would be a waste of time. Participating had changed her mind; however, and at the time of the interview, she described that it was very good and said the knowledge she had obtained was very useful for her work. Another RN described her participation as follows:

RN: I thought the intervention was very helpful; there were a lot of things you felt would be useful in your daily work as a telephone nurse. The questions we addressed in the intervention turn up now and then in conversations with patients, so it wasn’t one day too early to take part in this intervention. You could almost say it would’ve been useful to have done it a long time ago.

Interviewer: You have lacked this knowledge?

RN: Yes. (Interview 8)

Several RNs described having a need for more and recurrent education on the sick leave process. Suggestions for improvement included allowing a longer intervention time and including other professions as participants in the sessions. Many RNs used the concepts “learned more” or “got insight into” to describe the experience of participating in the intervention. Only a few used the concept “understanding” during the interviews. The experience of gaining increased knowledge was mainly described in five areas (sub‐categories): *Gaining knowledge about rules and regulations, Gaining knowledge of actors' different roles, Gaining knowledge of the patient's perspective of sick leave, Learning from peers in group discussions and Learning by reflecting and asking questions.*

#### 4.2.1 Sub‐category: Gaining knowledge of rules and regulations

RNs described that the intervention had given them knowledge about the rules and regulations concerning Swedish social insurance and about the different actors involved in the process. They also described gaining knowledge of the different time limits (*Rehabiliteringskedjan*) attached to the patients’ sick leave process. For many RNs, this was previously unknown and the knowledge was perceived as very useful for their understanding of the sick leave process.

#### 4.2.2 Sub‐category: Gaining knowledge of actors' different roles

Another area of knowledge involved actors’ different roles in the sick leave process, both within and outside the workplace. It was described as positive, in that it gave RNs an overview of what different professionals and actors do. Knowledge of the physician’s role when issuing sickness certificates and the role of the Swedish Social Insurance Agency were described as especially useful for the RNs seeking to understand the sick leave process. One RN described that, thanks to this knowledge, she now understands why the Swedish Social Insurance Agency sometimes asks for clarification of a sickness certificate. It also helped the RN to understand why patients sometimes called for help with issues like incomplete certificates and that part of the RN’s role was to support them in their needs.

#### 4.2.3 Sub‐category: Gaining knowledge of the patient’s perspective of sick leave

A deepened understanding of sick‐listed patients’ situations and especially their vulnerability was also experienced by some RNs as a result of the intervention. One RN described how, during one of the lectures, she came to realize that not everyone who is sick‐listed wants to be at home; she now thinks differently about this. Thus, the intervention was described as having changed her preconceived ideas about patients on sick leave.

#### 4.2.4 Sub‐category: Learning from peers in group discussions

The RNs also gained increased knowledge by discussing SLQs with other participants in the intervention’s group discussions. One RN described the sharing experience as follows: “It actually feels really good; it’s helpful to meet in a group like this. To give and take” (Interview 12). The RN further described that this created a sense of pride in being part of a larger context working towards the same goals. Hearing from other participants about different workplaces’ varying solutions for the same kinds of problems was also regarded as positive element of the education and added to their knowledge:

That we got to discuss the questions in a group and could listen to each other’s experiences, because we have slightly different experiences depending on which health care centre we work at; there are different patient groups and at some places there are more patients who call in for an extension of their sick leave because they’ve been excluded from social insurance and have to start over from the beginning. There were nurses with very different experiences and it gave me a lot to discuss with others. (Interview 7)

#### 4.2.5 Sub‐category: Learning by reflecting and asking questions

Some of the RNs also described an experience of learning how to handle different questions about sick leave: How to think and what questions to ask the caller. This was also described as very useful knowledge, as it can easily be applied in telephone calls. The reflection assignment was highlighted to give participants the opportunity to identify actual cases of their own with SLQs to reflect on. Some of the RNs did their assignments with other professionals at their workplace, which allowed them to discuss local routines.

### 4.3 Domain: Experiences of changes in handling sick leave questions

The domain *Experiences of the intervention* was described with the category *Having skills to handle SLQs* and sub‐categories *Knowing what to say and do* and *Knowing where to turn for support*.

### 4.4 Category: Having skills to handle sick leave questions

This category includes descriptions of attaining skills to handle SLQs by telephone. The interviewed RNs described that by participating in the intervention they acquired the skills to handle SLQs. Those skills are described in two sub‐categories: *Knowing what to say and do* and *Knowing where to turn to get support*.

#### 4.4.1 Sub‐category: Knowing what to say and do

The most prominent change in handling SLQs after the intervention was that most of the RNs described that they had changed their approach to communications with callers, due to now knowing what to say and do. As they now know, to a greater extent, what to say and do, the RNs perceived their assessments to be easier and faster, which created a feeling of comfort and confidence in their role as telephone nurses when handling SLQs. Some also noted that it feels good to know more than the patient does. Before the intervention, the RNs had sometimes experienced patients who knew more about the sick leave process than they themselves did, which generated unpleasant feelings in the RNs. The deepened knowledge resulted in the RNs being able to give callers more help than before, which was reported to contribute to their increased job satisfaction. The attained knowledge also made the RNs able to discuss with the callers their future in relation to the sick leave and allowed them to coach and encourage them to, for example, try out a previously discussed part‐time sick leave. One RN mentioned that the intervention had made her more attentive to the risk factors involved with long‐term sick leave. She now believed that she could take action to prevent sick leave by piloting the patient to a physiotherapist while they waited for an appointment with an orthopaedic specialist:

Interviewer: In what way has the educational intervention in social insurance medicine changed the way you work with sick leave questions?

I11: You feel more secure in your role

Interviewer: If you were to specify or tell me a bit more about that – what do you mean?

I11: My own experience – that I know how the situation is today – they demand…when it comes to a doctor’s certificate or sick pay, that they can call and say they need the certificate…they can call after three days and say I need to be on sick leave because I’m supposed to start working again next week but I don’t feel like I’m able to. You’ve always had the right to one week.

Interviewer: But they call already after three days and that's not even half the time. What do you do then?

I11: I explain that you have the right to stay home for a week and starting on the eighth day you have to call again, and you have to call that day; and you have to keep in touch

Interviewer: You didn't do exactly the same thing before?

I11: You did it, but now you feel even more sure. (Interview 11)

#### 4.4.2 Sub‐category: Knowing where to turn for support

RNs described that after the intervention they knew where to turn to get support in their assessment when they did not know how to handle a telephone call. Several RNs stated that although they did not always know what to do, they had learnt where to turn to figure it out. One RN described that, instead of immediately making an appointment with a physician, she could now talk to the physician to get advice and then solve the problem herself by talking with the patient. This created a feeling of comfort and confidence, at being able to handle callers’ questions more independently. The rehabilitation coordinator, in particular, was appointed to give them more support than before, as they now understand the role of the coordinator. Thus, the intervention had made them more aware of the rehabilitation coordinator’s and other professionals’ possibility of providing help:

I6: After that, I realize I can explain to the patients if they call, how much time it takes and…does it work for me to see the doctor three days after my certificate has expired and things like that. So, I’ve learned to answer the questions, to address their worries. For instance, when the certificate expires it’s okay if three more days go by – you’ve been on sick leave such a long time it doesn’t matter. But it’s mostly that – that I can answer in another way than I could before. Before, I would make an appointment with the doctor for everything; now, I don’t need to do that. I know I can go to the rehabilitation coordinator – I didn’t know what she did before; or, I knew what she did, but I didn’t know I could go to her instead of the doctor. (Interview 6)

## 5 DISCUSSION

The main finding in the present study was that the intervention contributed to the RNs gaining role clarity in the work with SLQs. The RNs’ descriptions of what they learned (see sub‐categories in Table 3) correspond with the content of the intervention. Thus, the findings indicate that their increased knowledge came not only from participating in the lectures and the group discussions, but also from completing the reflection assignment.

When evaluating whether the intervention enhanced competence for the participating RNs, the ideas or Benner (1984), Dall’Alba & Sandberg (1996), Sandberg (2001) and Schön (1987, 1995 ) can be used. After the first half‐day of education, the RNs had the chance to apply their newly learned theoretical knowledge to problems in practice, which both Benner (1984) and Schön (1987, 1995 ) point out is a way for professionals to develop competence. Thus, the reflection assignment gave the RNs the opportunity to apply what they learned during first education session. Through the opportunity of describing a problem from practice and later discussing it with other participants and being coached by the educators, conditions were set to create a “reflective practicum” (Schön, 1987, 1995 ). This reflective practicum gave the RNs the opportunity to have their preconceived knowledge and expectations challenged, improved and/or falsified (Benner, 1984; Miraglia & Asselin, 2015).

After six months, most of the RNs described that the intervention had resulted in their taking more action during the telephone calls than they had done before. That is, they took on more of the tasks related to telephone nursing (collecting information; assessing care needs; making decisions; giving advice, support and education; referring to the optimal level of care; giving healthcare information; and coordinating care (Greenberg, 2009; Kaminsky, 2013; Rutenberg & Greenberg, 2012)) than they had previously. For example, many RNs described giving more *information* to the callers than before. Another example is an RN who described that understanding the vulnerability and conditions of patients on sick leave had changed how she viewed these patients. This most likely opened up the opportunity for her to take notice of patients’ suffering and to be more supportive in the telephone calls. Another RN described being able to discuss*,* coach and encourage a caller to try part‐time sick leave. Thus, for this RN, the intervention led to attaining a more health‐promotive attitude to the work. In previous studies with telephone nurses, the health‐promotive potential of telephone nursing has been described as a field with great potential for development (Kaminsky, 2013).

As previously mentioned, the interview study with RNs indicated that work with SLQs can be seen in different ways. Some saw their role when handling SLQs as that of merely a “time booker,” while others took on a more independent way of working, as they did when dealing with other calls (Lännerström et al., 2012). This is in accordance with Sandberg’s (2001) thought that it is the understanding of the work that steers one’s actions while working. Some of the present study’s findings—RNs’ descriptions of offering increased support, coaching and information in telephone calls—are descriptions of changed actions. Thus, as the actions might have changed, it can be assumed that the understanding of the work also changed, when compared with the previous actions and due to the intervention.

Taking on a more independent role can be seen as a way of learning to *be* a more professional nurse (Dall’Alba & Sandberg, 1996; Sandberg, 2001). Being a professional nurse, as previously described, includes promoting health, preventing illness, restoring health and alleviating suffering by identifying and meeting the care‐seeker’s needs (Greenberg, 2009; Rutenberg & Greenberg, 2012). Applied to telephone nursing this means collecting information; assessing care need; making decisions; giving advice, support and education; referring to the optimal level of care; giving healthcare information; and coordinating care (Greenberg, 2009; Kaminsky, 2013; Rutenberg & Greenberg, 2012). After the intervention, some of the RNs described moving towards handling more professional telephone nursing tasks and thus moving towards being a professional RN.

One finding that seemed to give the RNs clarity regarding their own role involved the gained understanding of the roles of other professionals (physicians and the rehabilitation coordinator) and actors (the Swedish Social Insurance Agency) in the sick leave process. Understanding others’ roles seemed to help the RNs to achieve clarity regarding their own role. Previous research involving primary care teams concluded that defining others’ roles is a facilitator for developing one’s own role in collaborative work (Arksey, Snape, & Watt, 2007; Belanger & Rodriguez, 2008).

Previous research on RNs’ work with sick leave is sparse. The results of one study describing RNs’ experience participating in an educational intervention in social insurance medicine (Müssener & Linderoth, 2009) correspond with those of the present study. Both of these studies investigated educational interventions, and both had increased knowledge as an outcome. Another similarity between the studies was the RNs’ increasing willingness to make appointments with other professionals and their experience that, after the intervention, it was easier to awaken their patients' own sense of responsibility in the process.

### 5.1 Strengths and limitations

One of this study’s strengths is that a researcher uninvolved in the intervention conducted the interviews. This may have contributed to the participants’ answers being more truthful than if someone of the educators had conducted the interviews. However, there might be a risk that the participants’ answers were biased towards what they thought they were expected to say, a phenomenon known as the social desirability response bias (Polit & Beck, 2012). Although the interviews were performed six months after the intervention, it had made a lasting impression on the participants, who remembered what they had learned and gave examples of the effect it had had on their present daily work.

A limitation of the study is that it was a short intervention and held in only one county. The Swedish setting and healthcare organization coupled with the small sample are further limitations. However, the data were rich, and the RNs gave vivid examples of their experiences.

Different strategies were used to achieve trustworthiness (Lincoln & Guba, 1985). One was to increase the credibility of the interview data by involving a researcher in the interviews who had not been part of the education group. Another strategy was to have three researchers (TW, EK and IKH), none of whom had been part of the education and only one (LL) who had conducted the analysis. This, together with quotes in the *Findings* section, strengthens the study’s confirmability. Transferability of the findings to similar settings and samples, for example, other counties in Sweden educating RNs working with telephone nursing in primary health care, should be possible in terms of understanding the experience of participation. The study’s transferability also concerns the fact that a short education programme seemed to give effects (described by the participants), but this also needs to be tested further in a statistical evaluation of the intervention.

## 6 CONCLUSION AND CLINICAL IMPLICATIONS

This short educational intervention was perceived to increase RNs’ knowledge of the Swedish sick leave process and skills in handling SLQs in telephone nursing. In doing this, RNs gained role clarity in their work with SLQs. All parts of the intervention, including the lectures, discussions and reflection assignment, were described by participants as useful for their present work.

These findings are usable for healthcare professionals working to make quality improvements to the sick leave process and can serve as an example of how short educational interventions can contribute to experienced changed ways of working in practice. RNs’ work with SLQs is a new research area that needs further exploration. Future studies should investigate how patients are affected by RNs’ enhanced competence and how other professionals engaged in the sick leave process experience the RN’s role and competence.

## CONFLICT OF INTEREST

The authors declare no conflict of interest.

## AUTHOR CONTRIBUTIONS

LL, TW and IKH: Study design. EK: Conducted the interviews. LL and IKH: Performed the initial analysis. All authors then discussed and consented on the analysis. LL: Manuscript drafting, and all authors have approved its final version and meet at least one of the following criteria [recommended by the ICMJE (https://www.icmje.org/recommendations/)]:

- substantial contributions to conception and design, acquisition of data or analysis and interpretation of data;
- drafting the article or revising it critically for important intellectual content.
